# ACADL and ADH1B signify ketone body metabolic reprogramming in osteoarthritic synovium: insights from bioinformatics and animal model studies

**DOI:** 10.3389/fmed.2026.1700784

**Published:** 2026-02-03

**Authors:** Kuokuo Li, Bingshu Chen, Xun Yang, Yijun Yuan, Siyao Yang, Jinteng Liu, Jiawei Guo, Meng He

**Affiliations:** 1 Department of Traumatic Orthopedics, Shenzhen Second People’s Hospital (The First Affiliated Hospital of Shenzhen University), Shenzhen, China; 2 Department of Hand and Foot Surgery, Shenzhen Second People’s Hospital (The First Hospital Affiliated to Shenzhen University), Shenzhen, China; 3 Shenzhen University Medical School, Shenzhen University, Shenzhen, China; 4 Division of Adult Joint Reconstruction and Sports Medicine, Department of Orthopedic Surgery, Shenzhen People’s Hospital (The First Affiliated Hospital, Southern University of Science and Technology, The Second Clinical Medical College, Jinan University), Shenzhen, China; 5 Department of Medical Record, Shenzhen Second People’ s Hospital (The First Hospital Affiliated to Shenzhen University), Shenzhen, China; 6 Jinan University—University of Birmingham Joint Institute, Guangzhou, China; 7 Guangdong Key Laboratory for Biomedical Measurements and Ultrasound Imaging, National-Regional Key Technology Engineering Laboratory for Medical Ultrasound, School of Biomedical Engineering, Shenzhen University Medical School, Shenzhen, China

**Keywords:** osteoarthritis, ketone bodies, biomarkers, inflammation, molecular conformation

## Abstract

**Introduction:**

Osteoarthritis (OA) is characterized by articular degeneration and chronic joint pain, partly resulting from synovial inflammation. Accumulating evidence suggests that alterations in the synovial ketone body metabolism (KBM) are closely associated with OA pathogenesis. This study aimed to investigate the metabolic changes in synovial tissues to optimize the treatment of clinical OA.

**Methods:**

Analysis of OA and normal control synovial transcriptomic datasets extracted from the Gene Expression Omnibus (GEO) identified 808 differentially expressed genes (DEGs). These DEGs were integrated with KBM-related genes from the metabolic databases, yielding 50 candidates related to OA progression. Following enrichment analysis, protein-protein interaction network construction via STRING, and machine learning with expression analysis, two genes were identified as OA biomarkers: ACADL, encoding long-chain acyl-CoA dehydrogenase and *ADH1B*, alcohol dehydrogenase 1 B.

**Results:**

The nomogram based on these data revealed high accuracy in the training and validation sets. Functional analysis revealed that these genes function in lipid oxidation, a process critical for synovial cell energy metabolism, as well as in the redox balance that prevents oxidative stress from worsening OA inflammation. Immune infiltration analysis revealed that their expression significantly correlated with 21 immune subtypes, including pro-inflammatory M1 macrophages and Th17 cells, which drive synovial inflammation. Molecular docking analysis identified progesterone and fomepizole as potential agents with satisfactory affinities for *ACADL* and *ADH1B*, respectively. Assessment of mouse models of OA confirmed a significant reduction in the synovium at the protein level.

**Discussion:**

*ACADL* and *ADH1B* link KBM abnormalities to immune dysregulation in the OA synovium. The nomogram enables the precise early diagnosis of OA, and progesterone and fomepizole are promising targeted therapies. These findings deepen the current understanding of OA pathogenesis and support the advancement of personalized treatments for clinical translation.

## 1 Introduction

Osteoarthritis (OA) is a chronic joint disease that occurs among individuals worldwide. The latest data from the Global Burden of Disease indicate that OA affected 7.6% of the global population in 2020, with its prevalence variably projected to increase by 132.2% over the next three decades and by 60es a% by 2050 (1). OA is one of the most common causes of chronic pain and disability among the elderly (2). Pathologically, OA is characterized by cartilage degradation and synovial inflammation, ultimately leading to joint dysfunction (3, 4). Clinically, management of early-to mid-stage OA includes weight loss, physical therapy, analgesic medications, and chondroprotective agents to alleviate joint symptoms. Conversely, in advanced OA, surgical interventions, such as osteotomy and joint replacement are required (5). Insufficient understanding of the molecular mechanisms underlying OA pathogenesis has rendered disease progression irreversible, with current strategies limited to symptomatic treatments (6). Therefore, exploring the aberrant molecular signatures in OA tissues may facilitate the identification of therapeutic targets that influence disease progression, consequently promoting the development of etiological interventions.

Arthroscopic and imaging examinations of affected patients have revealed that approximately 50% of patients with OA with knee pain presented with synovitis, with its severity positively correlated with the degree of pain (1, 7, 8). Infiltrated macrophages and T lymphocytes can release interleukin-1β, tumor necrosis factor-α, prostaglandin E2, and nerve growth factor. These factors induce peripheral and central sensitization, ultimately contributing to pain (9). Reciprocally, they can interrupt local synovial cells during lipid metabolism, which is involved in the alternative energy supply and inflammatory cytokine synthesis (10, 11), creating a vicious cycle driving disease progression.

Specifically, ketone bodies generated from fatty acid β-oxidation under stress conditions play core roles in both metabolic reprogramming and cellular signaling. Studies have demonstrated that elevated blood ketone body levels ameliorate inflammation and cartilage loss in rat models of OA (12). A recent study further confirmed that β-hydroxybutyrate enhances chondrocyte mitophagy via the HCAR2/AMPK/PINK1/Parkin pathway, thereby reducing chondrocyte senescence and inflammatory cytokine secretion (13). Consequently, we hypothesized that aberrant ketone body metabolism (KBM) in the synovial cells induces inflammation and contributes to joint pain.

This study aimed to integrate transcriptomic datasets, employing bioinformatics approaches to comprehensively identify KBM-associated genes in the synovium of patients with OA, elucidate their biological implications, and validate the findings in animal models. Ultimately, this study provides a novel theoretical foundation for deciphering the KBM-related mechanisms in OA and formulating targeted therapeutic strategies.

## 2 Materials and methods

### 2.1 Data collection

Gene expression profiles of normal and OA synovial tissues were obtained from the Gene Expression Omnibus (GEO) database.^1^ The GSE55235 dataset (platform: GPL96) was selected as the training cohort, which comprised transcriptomic data from 10 OA samples and 10 healthy controls. For validation purposes, the GSE82107 dataset (platform: GPL570) containing 10 OA and seven healthy synovial tissue transcriptomes was retrieved from the same repository. KBM-related genes were obtained from the Molecular Signatures Database (MSigDB)^2^ using the keywords “Ketone Body” and “homo sapiens.” After removing duplicates, 485 unique KBM-related genes were retained for subsequent analysis (Supplementary Table 1).

### 2.2 Differential expression analysis

Identification of DEGs between OA and control samples from the GSE55235 training set was achieved using the “limma” R package (v 3.54.0) (14). Genes with an adjusted *p* < 0.05 and an absolute log_2_Fold Change (| log_2_FC|) > 1 were considered statistically significant. The Benjamini-Hochberg method was employed for *p*-value adjustment. A chart displaying the top 10 genes with the highest dysregulation (arranged by decreasing | log_2_FC| values) was generated with the R package “ggplot2” (v 3.4.1) to visualize expression patterns (15). Expression profiles of the top 25 up-regulated and down-regulated DEGs were mapped using the R package “pheatmap” (v 1.0.12) (16).

### 2.3 Candidate genes acquisition and functional enrichment analysis

For the purpose of identifying genes related to ketone body metabolism, a Venn diagram analysis was performed utilizing the R package “ggvenn” (v 0.1.9) (17) to determine overlapping genes between the two gene sets, and the intersection genes of the two partial genes were defined as candidate genes for subsequent analysis.

To delve into the biological processes and signaling mechanisms that contribute to the advancement of OA disease, enrichment analyses for Gene Ontology (GO) and Kyoto Encyclopedia of Genes and Genomes (KEGG) pathways were carried out on the candidate genes set employing the R package “clusterProfiler” (v 4.7.1.3) (18). Statistical significance was set at *p* < 0.05. The top 10 terms with the lowest *p*-values were depicted using the R package “enrichplot” (v 1.18.3) (19).

### 2.4 Protein-protein interaction network

To understand the interaction between proteins encoded by candidate genes, PPI network was constructed for candidate genes, candidate genes were inputted into the STRING database^3^ with a minimum interaction confidence score set at 0.4. The PPI network was then constructed using standard parameters and subsequently visualized using Cytoscape software (v 3.8.2) (20).

### 2.5 Feature gene selection using machine learning

To further refine candidate genes, two independent machine learning algorithms-Boruta analysis and eXtreme Gradient Boosting (XGBoost)-were applied to screen feature genes in the training set (OA vs. Control samples). The Boruta algorithm, a random forest-based global feature selection method, was implemented using the R package “Boruta” (v 8.0.0) (21). This method evaluates the relative importance of all candidate genes by comparing their significance with randomly permuted “shadow features.” Genes with importance scores statistically higher than shadow features (*p* < 0.05) were retained as significant features. The XGBoost algorithm, an optimized gradient-boosting framework, was employed through the R package “XGBoost” (v 2.1.1.1) (22). Gene importance was ranked based on the “gain” metric, which quantifies the contribution of each gene to model accuracy improvement. The Venn diagram was produced with the aid of the R package “Venn Diagram” (v 1.7.3) (23) to identify overlapping genes derived from Boruta and XGBoost. Genes common to both methods were defined as final feature genes for subsequent validation and functional studies.

### 2.6 Identification of biomarker

To further pinpoint and confirm the expression of characteristic genes, a Wilcoxon rank test was conducted on disease and control samples in the training set (GSE55235) and the validation set (GSE82107). A two-tailed *p* < 0.05 was considered statistically significant. The expression levels of the final feature genes (derived from the intersection of Boruta and XGBoost) were compared between OA and control groups. Biomarkers were characterized as genes that display statistically significant differential expression (*p* < 0.05) and concordant trends across both the training and validation datasets.

### 2.7 Construction and validation of the clinical nomogram

In order to elevate clinical applicability, a nomogram was developed employing the R package “rms” (v 6.7.0) (24) based on the biomarkers identified in previous analyses. In this model, “Points” represented individual scores assigned to each gene, while “Total Points” denoted the cumulative score derived from all selected genes. The total score was utilized to predict OA prevalence. Calibration curve: the nomogram’s predictive accuracy was evaluated employing the R package “regplot” (v 1.1) (25). A calibration plot comparing predicted probabilities with observed outcomes was generated, where a slope close to 1 indicated high model reliability. Receiver Operating Characteristic curve analysis: the nomogram’s discriminative power was confirmed through the utilization of the R package “pROC” (v 1.18.0) (26). Area under the curve > 0.7 was considered indicative of robust predictive performance.

### 2.8 Functional analysis of biomarkers

Gene Set Enrichment Analysis (GSEA) was executed to delve into the biological functions of the identified biomarkers in the development of OA. The steps taken in the analysis included: Performing Spearman correlation analysis between each biomarker and all other genes in the OA training set (GSE55235) with the aid of the R package “psych.” (v 2.1.6) (27). Genes were ranked based on correlation coefficients (from highest to lowest). The gene list was analyzed through GSEA employing the R package “clusterProfiler” (version 4.6.2) (28), in conjunction with the KEGG pathway gene set (c2.cp.kegg.v7.0.entrez.gmt) from the MSigDB repository.^4^ Enrichment was deemed significant when it met the criteria of a nominal *p* < 0.05, a false discovery rate (FDR) < 0.25, and an absolute normalized enrichment score (| NES|) > 1. The five pathways with the highest enrichment scores, were graphically represented using the R package “enrichplot” (v 1.18.3) (19).

In order to further evaluate the interactions between biomarkers and their functionally similar genes and related functions, the GeneMANIA database^5^ was utilized. The analysis followed these steps: biomarkers identified in previous analyses were submitted to GeneMANIA. GeneMANIA’s built-in algorithms assigned weights to interaction types based on their relevance to the input genes, and functionally similar genes were retained for network visualization.

### 2.9 Immune infiltration analysis

The xCell algorithm was used to assess the infiltration levels of 34 immune cell types in the OA training set (GSE55235) employing the R package “xCell” (v 1.1.0) (29). Heatmaps and boxplots were generated to visualize differential immune infiltration patterns between OA patients and normal controls. Wilcoxon rank-sum tests were conducted employing the R package “stats” (v 4.2.2) (30) to contrast immune cell infiltration scores between the OA and control groups. Immune cell types with significantly altered infiltration levels (*p* < 0.05) were designated as differentially infiltrated immune cells. Boxplots illustrating these differences were created using the R package “ggplot2” (v 3.4.1) (15). Spearman correlation analysis between differentially infiltrated immune cells was conducted across all training set samples using the R package “psych” (v 2.1.6) (27), the correlation coefficients (| cor| > 0.3) and (*p* < 0.05) were considered significant. Additionally, the package was utilized to analyze the Spearman correlation between biomarkers and differentially infiltrating immune cells (| cor| > 0.3, *p* < 0.05), and the correlation results were visualized using a heatmap.

### 2.10 Drug prediction and molecular docking analysis

To identify potential therapeutic agents targeting the biomarkers for OA treatment, the Drug-Gene Interaction Database^6^ was queried to retrieve drug-gene interactions. Drug-gene interaction networks were constructed and visualized using Cytoscape software (v 3.9.1) (20). Subsequently, the Comparative Toxicogenomics Database^7^ was utilized to obtained drugs associated with OA. Drugs overlapping between Drug-Gene Interaction Database and Comparative Toxicogenomics Database predictions were prioritized. OA-relevant drugs targeting biomarkers were selected for further molecular docking analysis. Their chemical structures were downloaded from the PubChem database.^8^ Molecular docking was performed using CB-Dock2.^9^

### 2.11 Animal model validation

We adopted surgery-induced OA mice model in this study, which was established by destabilization of the medial meniscus (DMM). 18 mice (C57BL/6, aged 8-week male, weighed ∼20 g) were purchased, acclimatized for 1 week under standard housing conditions (specific pathogen free, 22°C with a 12 h light/dark cycle and free access to standard rodent chow and water), and then randomly divided into two groups. For the OA group, we anesthetized the mice and made a 5 mm longitudinal incision on the medial side of the knee joint, then exposed the joint capsule. Next, we gently everted the patella to visualize the medial meniscus and its tibial attachment. Then we precisely transected the medial meniscal-tibial ligament, confirmed by slight meniscal displacement. Then we closed and disinfected the wound. For the control group, we performed the same procedures except meniscus destabilization. The success of the surgically induced model was confirmed 8 weeks post-surgery using behavioral and histopathological assessment (data not shown) (31).

For immunohistochemistry staining, we harvested 12 knee joints from the OA and control group, respectively. They were decalcified in 10% ethylenediaminetetraacetic acid (pH 7.4) at 37°C for 72 h with daily solution change prior to paraffin embedding. 5μm-thick slides were baked at 60°C for 2 h, deparaffinized in xylene, rehydrated through graded ethanol, and rinsed in distilled water. Antigen retrieval was conducted by microwave heating in 0.01 M citrate buffer (pH 6.0) (boiling for 5 min, simmering for 15 min), followed by cooling and phosphate-buffered saline washes. Endogenous peroxidase was blocked with 3% H^2^O^2^ (15 min), and non-specific binding with 5% bovine serum albumin (30 min). Then the slides were incubated with ACADL polyclonal antibody (Proteintech, Wuhan, China, 17442-1-AP, 1:200) or ADH1B polyclonal antibody (Affinity, Liyang, China, DF12809, 1:50) overnight at 4°C, followed by Goat anti-rabbit IgG H&L (HRP) secondary antibody (Abcam, Cambridge, UK, ab6721, 1:1,000) (60 min at room temperature). Signals were visualized with 3,3’-diaminobenzidine tetrahydrochloride (Beyotime, Shanghai, China, P0203), nuclei counterstained with hematoxylin, and sections dehydrated, cleared, and mounted. Positive cell proportion was calculated using ImageJ software.

For Western blot, we harvested 6 knee joints from the other mice of the two groups. Frozen synovial tissue was first weighed, minced, and homogenized on ice in radio-immunoprecipitation assay buffer lysis buffer containing 1% protease and phosphatase inhibitors; the homogenate was incubated on ice for 30 min, then centrifuged at 12,000 g for 15 min at 4°C to collect the supernatant. Protein concentration was quantified by bicinchoninic acid assay, and equal amounts of protein (30μg) were denatured by boiling with 5 × sodium dodecyl sulfate—polyacrylamide gel electrophoresis loading buffer at 95°C for 10 min. Samples were then subjected to above electrophoresis, followed by transfer to polyvinylidene fluoride membranes. The membranes were blocked with 5% skim milk in Tris-buffered saline with Tween-20 (TBST) for 60 min at room temperature, incubated with ACADL polyclonal antibody (Proteintech, Wuhan, China, 17442-1-AP, 1:1,000) or ADH1B polyclonal antibody (Affinity, Liyang, China, DF12809, 1:2,000) or Vinculin monoclonal antibody (Proteintech, Wuhan, China, 66305-2-Ig, 1:10,000) overnight at 4°C, washed with TBST, then incubated with Goat anti-rabbit IgG H&L (HRP) secondary antibody (Abcam, Cambridge, United Kingdom, ab6721, 1:10,000) or Rabbit anti-mouse IgG H&L (HRP) secondary antibody (Abcam, Cambridge, United Kingdom, ab6728, 1:20,000) for 60 min at room temperature. After final TBST washes, Signals were visualized with 3,3’-diaminobenzidine tetrahydrochloride (Beyotime, Shanghai, China, P0203) to be evaluated.

### 2.12 Statistical analysis

Bioinformatics analyses were conducted with the aid of the R programming language (v 4.2.2). The Wilcoxon rank sum test or non-paired Student’s *t*-test were applied to contrast the differences between two groups. Fisher’s exact test was used to compute the significance *p*-value. *p* < 0.05 was deemed statistically significant.

## 3 Results

### 3.1 Acquisition of 50 candidate genes for OA

**FIGURE 1:**
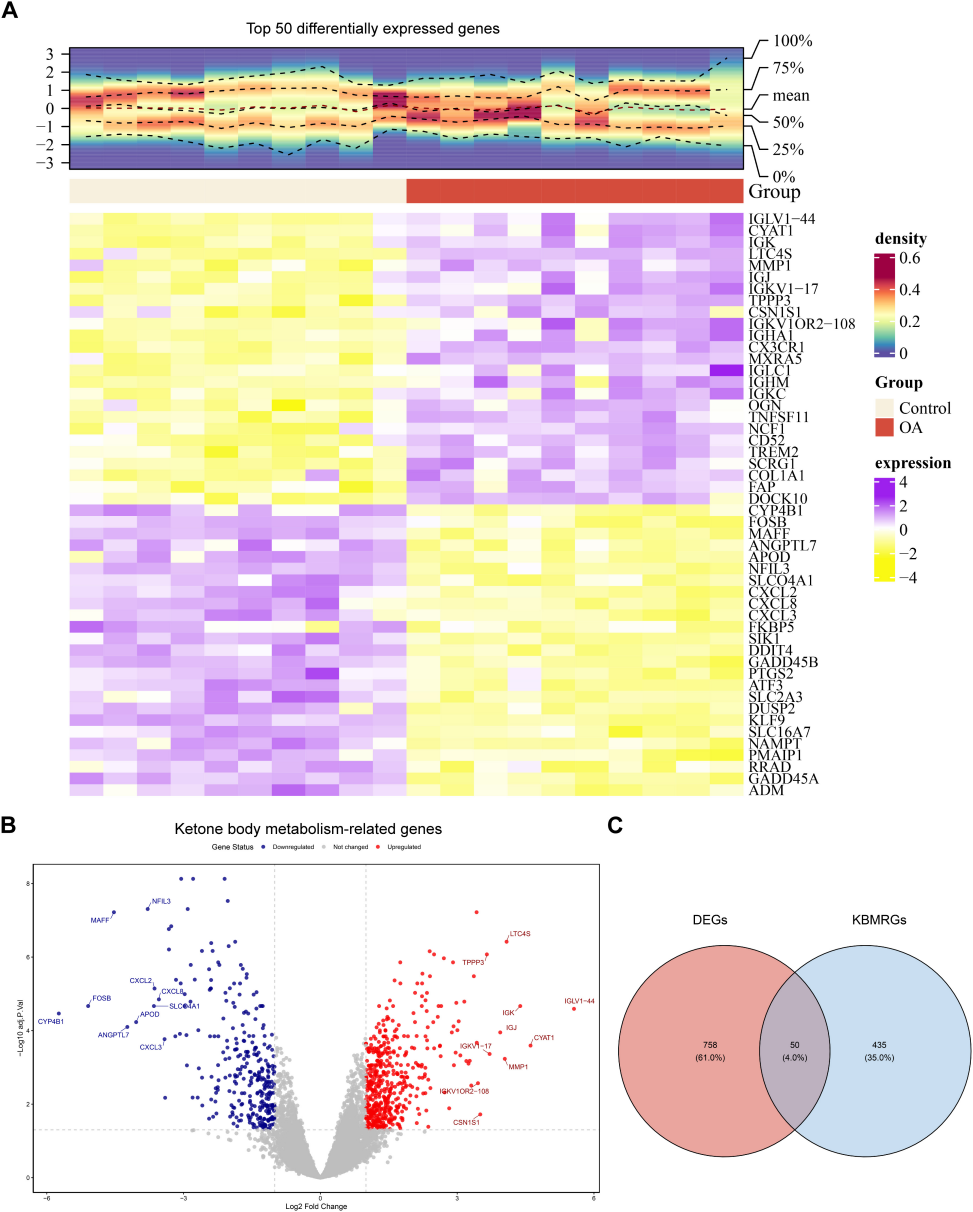
Candidate gene acquisition of ketone body metabolism in osteoarthritic synovium. **(A)** Heatmap of top 50 differentially expressed genes in the transcriptomic data from the osteoarthritic and control synovium. The color of the upper density heatmap represents the gene expression density of each sample, with redder colors indicating higher density; in the lower heatmap, the vertical axis represents genes, where purple denotes high expression and yellow denotes low expression. **(B)** Volcano plot of DEGs between OA and control groups (adjust *p* < 0.05 and | log2FC| > 1). **(C)** Venn diagram of the intersection between KBM-related genes and DEGs.

Transcriptomic analysis revealed 808 DEGs in OA samples in contrast to controls (| log_2_FC| > 1, adjusted *p* < 0.05), with 496 genes upregulated and 312 downregulated (Figure 1A). Subsequent intersection analysis with known KBM-related genes revealed 50 differentially expressed KBM-related genes (Figures 1B,C). These overlapping genes demonstrated significant dysregulation patterns, suggesting their potential involvement in OA pathogenesis.

### 3.2 Exploration of the functions and pathways associated with the candidate genes

To elucidate the biological roles of 50 candidate genes in OA pathogenesis, comprehensive functional annotation was performed. GO analysis revealed significant enrichment in 1,078 biological processes, 24 cellular components, and 104 molecular functions (Supplementary Tables 2–4). The top five biological processes terms were cellular ketone metabolic process, regulation of cellular ketone metabolic process, regulation of small molecule metabolic process, regulation of cellular ketone metabolic process, regulation of lipid metabolic process, and fatty acid metabolic process. These findings indicated the candidate genes’ predominant involvement in systemic metabolic regulation and lipid homeostasis.

**FIGURE 2:**
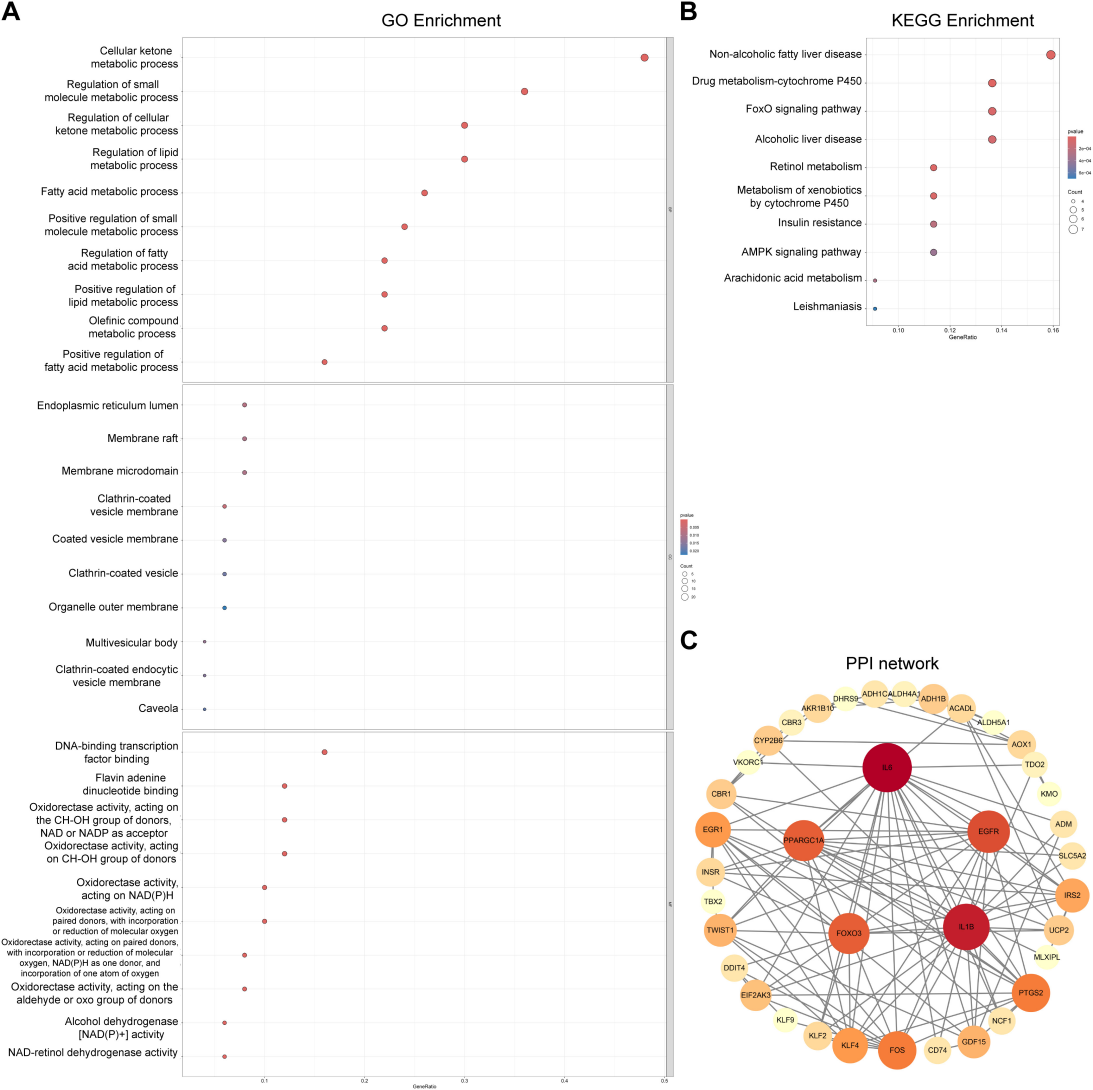
Function and interaction analysis of the candidate genes. **(A)** The GO enrichment analysis results indicated that the candidate genes are primarily involved in the regulation of systemic metabolism and lipid homeostasis (*p* < 0.05). **(B)** KEGG enrichment of the candidate genes (*p* < 0.05). **(C)** PPI network of the candidate genes (confidence score > 0.4).

Cellular components analysis highlighted enrichment in endoplasmic reticulum lumen, membrane raft, membrane microdomain, clathrin-coated vesicle membrane and coated vesicle membrane, suggesting their roles in vesicle-mediated transport and subcellular metabolic compartmentalization. Molecular functions analysis identified DNA-binding transcription factor binding, flavin adenine dinucleotide binding and oxidoreductase activities as dominant features and encoding NAD (P)-dependent enzymes played a critical role in redox balance maintenance during cartilage degeneration (Figure 2A).

KEGG pathway analysis further identified 62 significantly enriched pathways (Supplementary Table 5). The top five pathways, non-alcoholic fatty liver disease, drug metabolism-cytochrome P450, foxO signaling pathway, alcoholic liver disease, and retinol metabolism-highlighted crosstalk between local joint metabolism and systemic energy dysregulation (Figure 2B).

The PPI network comprised 38 nodes (proteins) and 123 edges (interactions) with a confidence threshold of 0.4 (Figure 2C). Notably, Interleukin-6 was found to interact with multiple proteins, including ACADL, EGR1, PPARGC1A, and FOXO3, highlighting its potential central role in the network. The dense interconnectivity indicated robust coordination among these genes. This structural organization suggested a tightly regulated metabolic cascade potentially disrupted in OA pathophysiology.

### 3.3 Identification of *ACADL* and *ADH1B* as biomarkers for OA

**FIGURE 3:**
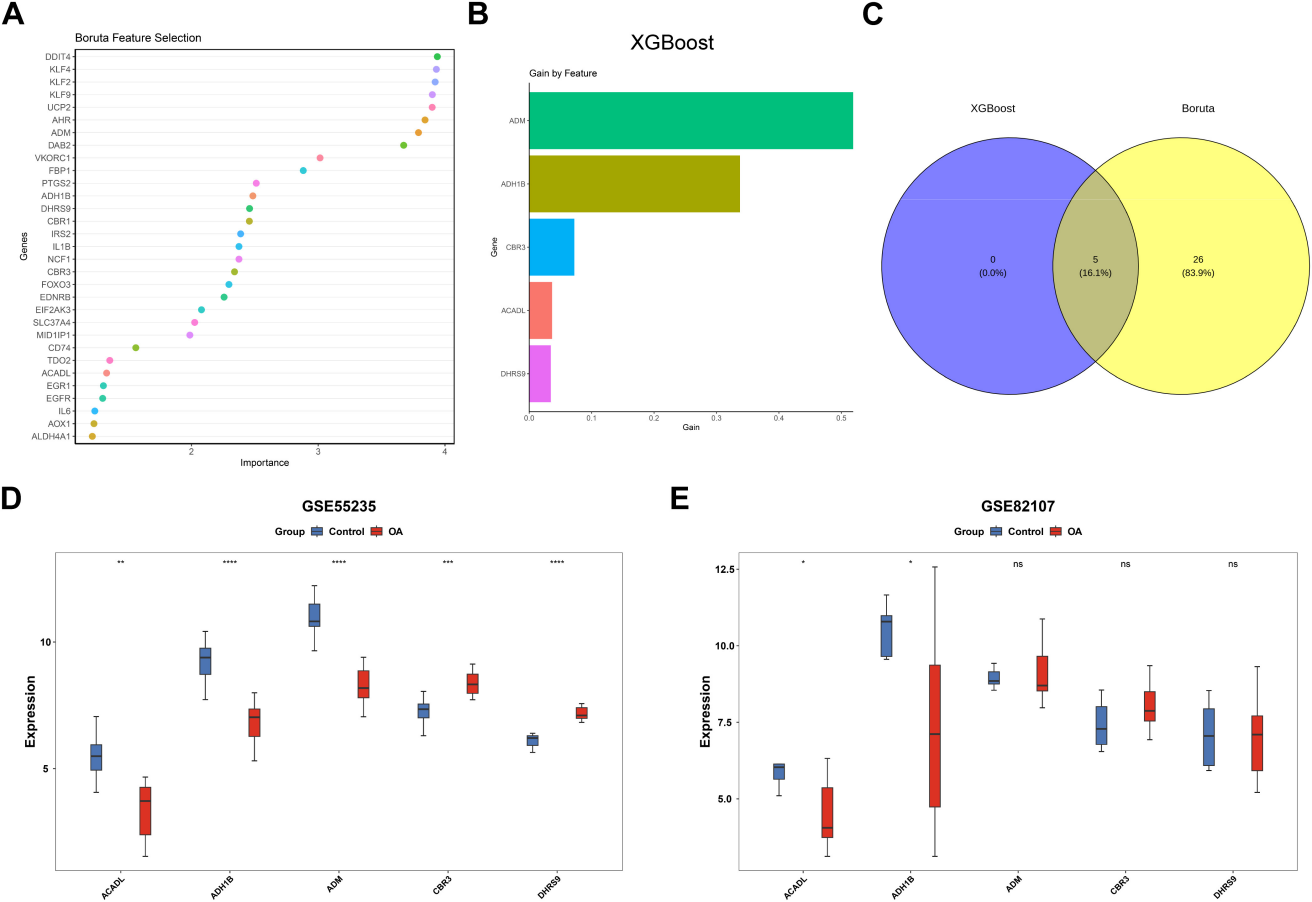
Two machine learning algorithms identified osteoarthritis biomarkers. **(A)** Based on the 31 feature genes screened by the Boruta algorithm, importance ranking was performed. **(B)** Based on the five feature genes selected by the XGBoost algorithm, the horizontal axis represents the contribution of each gene to the model’s gain, with larger values indicating a greater contribution to improving model performance. The vertical axis lists the names of the respective genes. **(C)** The intersection of the two algorithms identified five consensus genes. **(D,E)** Validations of the consensus genes in two independent cohorts (Wilcoxon rank-sum tests). Data were presented as Median with Q1, Q3 **(D,E)**. NS, not significant; *p < 0.05, **p < 0.01, ***p < 0.001, ****p < 0.0001.

Two machine learning algorithms were systematically employed to refine the candidate gene pool. The Boruta algorithm identified 31 feature genes (Figure 3A), while XGBoost identified 5 genes (Figure 3B). Intersection analysis revealed 5 consensus genes (*ADM, ADH1B, CBR3, ACADL, DHRS9*) shared by both methods (Figure 3C). Subsequent validation in two independent cohorts (GSE55235 and GSE82107) demonstrated significant downregulation of *ACADL* (*p* < 0.05) and *ADH1B* (*p* < 0.05) in OA samples compared to controls (Figures 3D,E). These findings position *ACADL* and *ADH1B* as critical molecular players in OA pathogenesis.

### 3.4 Clinical value of *ACADL* and *ADH1B*

**FIGURE 4:**
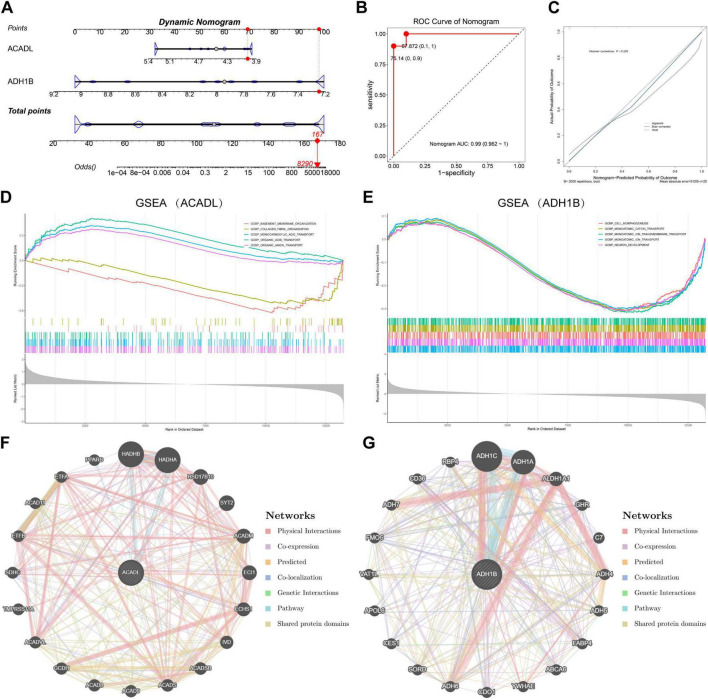
The evaluation of clinical predicting value, functional signaling pathways, and molecular crosstalk of *ACADL* and *ADH1B*. **(A)** The nomogram constructed based on the two biomarkers. “Points” indicates the individual scores corresponding to the values of each variable; “Total Points” represent the sum of all individual scores, which corresponds to the “Odds” axis at the bottom and can be used to estimate the predicted probability of the target event (e.g., disease occurrence). **(B,C)** The predictive performance of the nomogram model was jointly validated by ROC analysis **(B)** and calibration curves **(C)**, with results demonstrating its strong discriminative ability and calibration accuracy. **(D,E)** The primary pathways identified in GSEA of *ACADL* **(D)** and *ADH1B* **(E)**. **(F,G)** GeneMANIA-based interaction networks of *ACADL* **(F)** and *ADH1B* **(G)**.

A dynamic nomogram was developed to quantify OA progression risk, incorporating two biomarkers (*ACADL* and *ADH1B*). The model assigned differential point values to distinct expression ranges of each biomarker, enabling cumulative risk calculation through variable summation (Figure 4A). At a total score (total points = 167), the nomogram predicted an increase in OA progression odds (odds = 8,290). Receiver Operating Characteristic curve analysis achieved exceptional discriminative performance (Area under the curve = 0.99), maintaining diagnostic accuracy across validation cohorts (Figure 4B). Calibration curves revealed close alignment between predicted and observed OA probabilities (Hosmer-Lemeshow test, *p* = 0.229), confirming high prediction accuracy (Figure 4C).

### 3.5 Exploration of the functions and pathways associated with *ACADL* and *ADH1B*

GSEA was conducted to clarify the functional roles of *ACADL* and *ADH1B* in OA progression. Spearman correlation-based ranking revealed distinct pathway activation patterns for each biomarker. *ACADL* displayed a notable enrichment in 108 biological processes (*p* < 0.05, FDR < 0.25, | NES| > 1), as outlined in Supplementary Table 6. The primary pathways identified were organic anion transport, organic acid transport, monocarboxylic acid transport, basement membrane organization, and collagen fibril organization, as depicted in Figure 4D. These findings suggest *ACADL*’s dual involvement in metabolic transport and extracellular matrix remodeling, processes critical for cartilage homeostasis.

*ADH1B* demonstrated enrichment in 63 pathways (Supplementary Table 7), predominantly associated with ion homeostasis and neurodevelopmental processes. The most significant pathways included monoatomic ion transport, neuron development, cell morphogenesis, monoatomic cation transport, and ion transmembrane transport (Figure 4E). The enrichment of neurodevelopmental pathways implies potential crosstalk between OA-related metabolic dysregulation and sensory nerve remodeling in joint tissues.

To elucidate the functional cooperativity of *ACADL* and *ADH1B*, GeneMANIA-based interaction networks were constructed for both biomarkers (Figures 4F,G). The *ACADL*-centric network comprised 20 functionally associated genes, with physical interactions dominating the connectivity. Co-expression and predicted interactions constituted secondary connection modes, while co-localization, genetic interactions, pathway sharing, and domain homology contributed minimally. The *ADH1B* network similarly contained 20 interactors with identical edge-type distribution. Both networks displayed characteristic hub-and-spoke architecture, suggesting these biomarkers serve as critical nodes in their respective biological systems. The high prevalence of physical Interactions implied direct molecular binding between the biomarkers and their partners. These findings collectively highlight the central regulatory roles of *ACADL* and *ADH1B* in their corresponding metabolic pathways through extensive molecular interactions.

### 3.6 Correlation between *ACADL/ADH1B* and immune microenvironment in OA synovium

**FIGURE 5:**
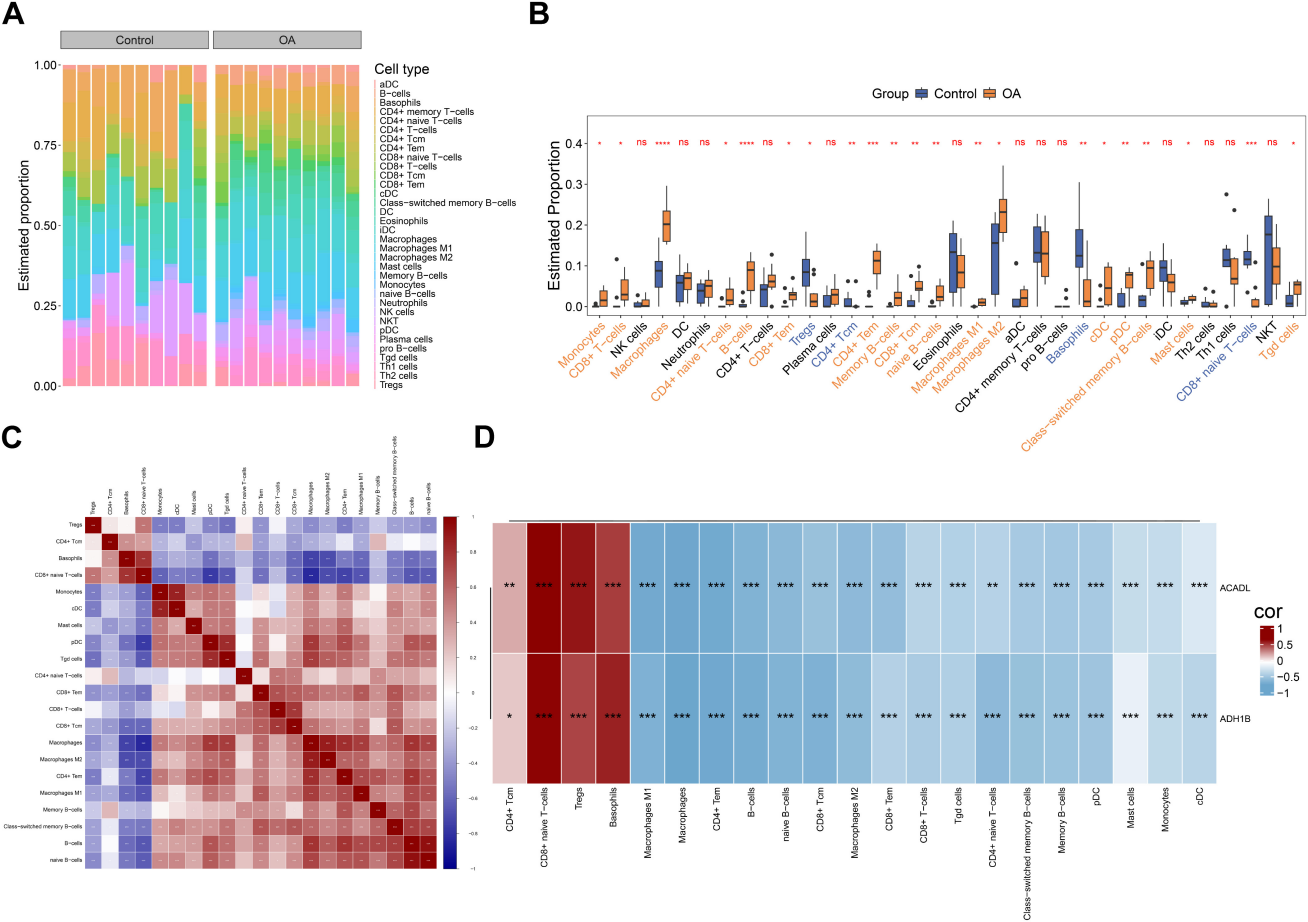
Immune microenvironment changes in OA synovium. **(A)** Heatmap of immune cell infiltration. **(B)** There exists a statistically significant difference in the infiltration levels of 21 immune cell types between the OA group and the control group (Wilcoxon rank-sum tests). **(C)** Correlation analysis among the 21 differential immune cell types (Spearman correlation analysis). **(D)** Correlation analysis between the biomarkers and differentially infiltrated immune cells (Spearman correlation analysis). Data were presented as Median with Q1, Q3 (B). NS, not significant; *p < 0.05, **p < 0.01, ***p < 0.001, ****p < 0.0001.

Comparative analysis of immune cell composition revealed distinct microenvironment alterations between OA and control groups. Stacked bar plots demonstrated differential distribution patterns of 34 immune cell subtypes across cohorts (Figure 5A). Immune infiltration analysis identified 21 immune cell subtypes with significant differences in infiltration levels between the OA and control groups (*p* < 0.05) (Figure 5B). Macrophages and B cells exhibited the most pronounced proportional increase in OA samples (*p* < 0.0001).

To investigate the coordinated regulation of immune components in disease progression, Spearman correlation analysis was conducted to evaluate intercellular interactions among immune cell subtypes. Cross-cell-type correlation analysis revealed a robust positive association (cor = 0.86, *p* < 0.0001) between monocytes and cDC transcriptional profiles (Figure 5C and Supplementary Table 8), indicating biologically meaningful co-regulation. This strong correlation pattern suggested synchronized activation states and functional synergy between the two cell populations.

Further analysis revealed distinct correlation patterns between key biomarkers (*ACADL* and *ADH1B*) and immune cell infiltration. Both genes exhibited predominant negative correlations with most immune cell types (*p* < 0.01), particularly with macrophages (*ACADL*: cor = −0.76; *ADH1B*: cor = −0.77) and CD4^+^ Tem (*ACADL*: cor = −0.74; *ADH1B*: cor = −0.75). It is worth noting that paradoxical positive correlations were observed in certain immune cell subsets, such as regulatory T cells (Tregs; *ACADL*: cor = 0.60; *ADH1B*: cor = 0.46), basophils (*ACADL*: cor = 0.48; *ADH1B*: cor = 0.56) and CD8^+^ naive T cells (*ACADL*: cor = 0.65; *ADH1B*: cor = 0.64), with all correlations reaching statistical significance (*p* < 0.05) (Figure 5D and Supplementary Table 9).

These results collectively suggest that *ACADL* and *ADH1B* may function through cell type-specific interaction mechanisms, influencing disease pathogenesis via immune-microenvironment modulation.

### 3.7 Pharmacological prediction and molecular docking analysis

**FIGURE 6:**
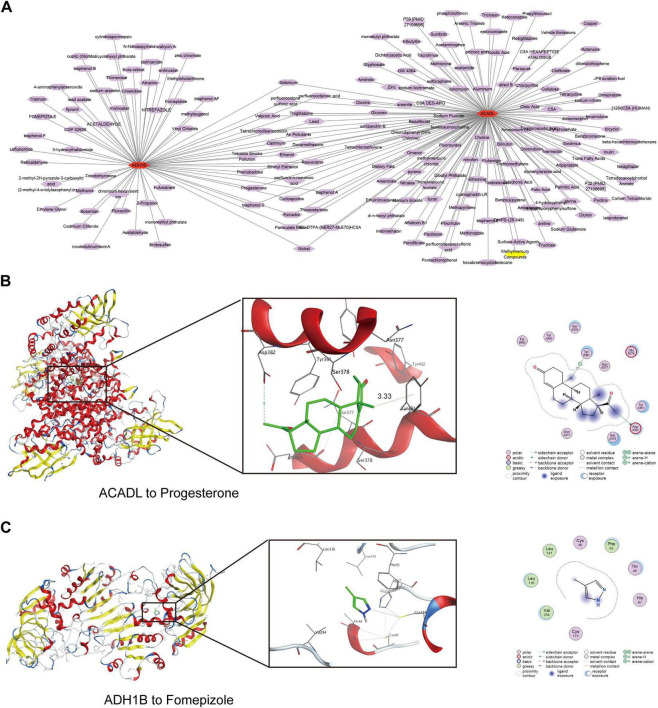
Pharmacological prediction and molecular docking of *ACADL* and *ADH1B*. (A) Drug-biomarker regulatory network. Orange nodes represent the two biomarkers, while purple nodes represent the predicted drugs. (B,C) Molecular docking simulations of *ACADL*-progesterone (B) and ADH1B-fomepizole (C).

A total of 192 drugs targeting two biomarkers (*ACADL* and *ADH1B*) were identified. Notably, elenium and nickel exhibited dual-targeting effects on both biomarkers. For *ADH1B*-specific targeting, fomepizole as prioritized, while progesterone was identified as the primary candidate for *ADH1B* and *ACADL* modulation (Figure 6A). Molecular docking simulations were performed to evaluate binding affinities between drugs and their targets. Progesterone demonstrated robust binding affinity with *ACADL* (docking score = −8.8 kcal/mol), while fomepizole exhibited strong interaction with *ADH1B* (docking score = −9.3 kcal/mol) (Figures 6B,C and Table 1).

**TABLE 1:** Molecular docking information of ACADL and ADH1B.

Protein	PDB ID	Compound	Compound CID	Docking score
ACADL	8W0T	Progesterone	5,994	−8.8 kcal/mol
ADH1B	1DEH	Fomepizole	3,406	−9.3 kcal/mol

### 3.8 Animal model validation for *Acadl* and *Adh1b* expression

**FIGURE 7:**
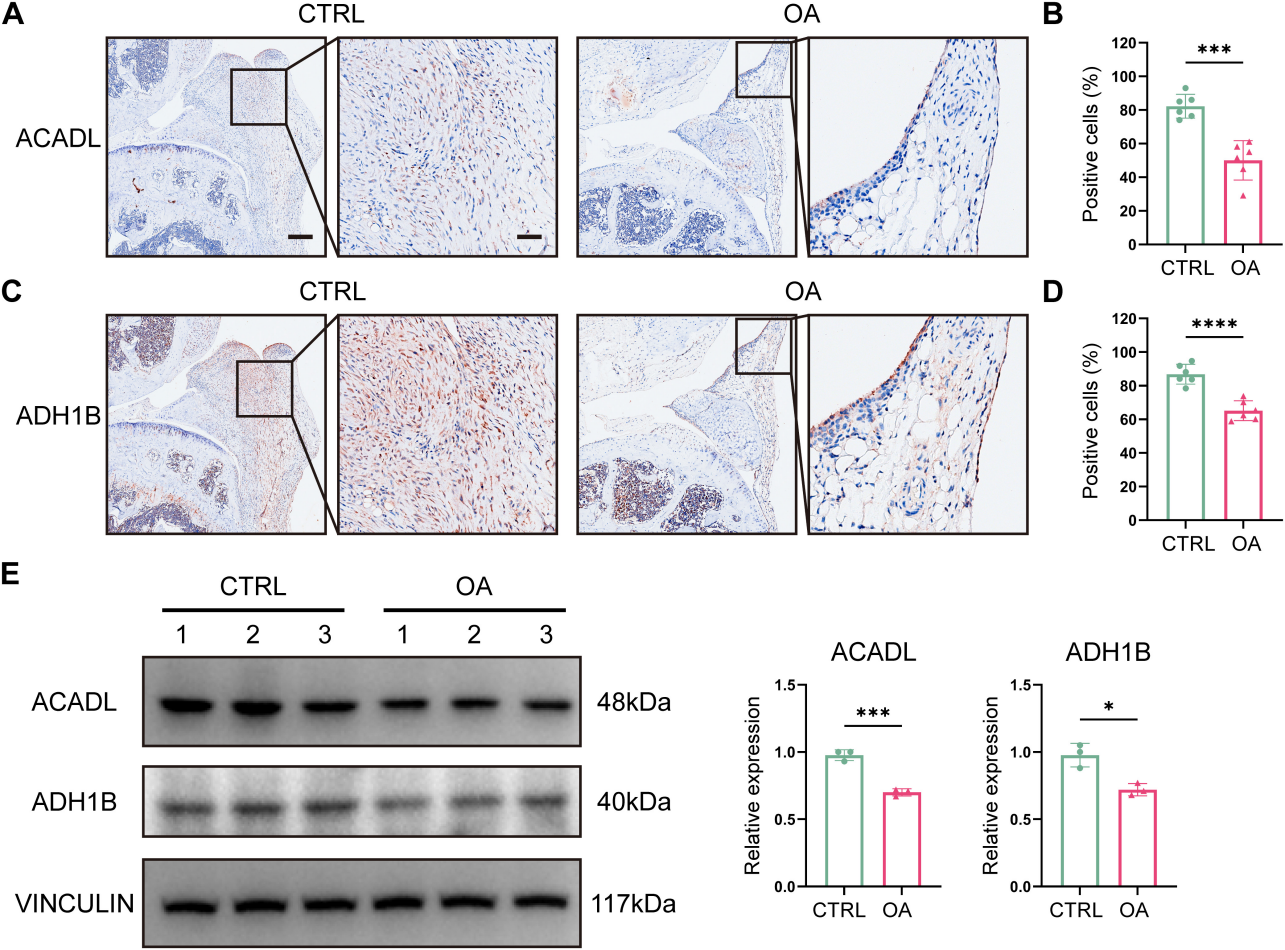
ACADL and ADH1B expression in murine synovium. (A–D) The representative images of immunohistochemistry staining of ACADL (A) and ADH1B (B) in the synovium of mouse model and statistical analysis. (E) Western blot analysis of ACADL and ADH1B of mouse model. CTRL, control; OA, osteoarthritis. Data were presented as Mean ± SD (B,D,E). *p < 0.05, ***p < 0.001, ****p < 0.0001, non-paired Student *t*-test. Scale bar = 200μm (left) or 50μm (right).

To confirm the expressional level of *Acadl* and *Adh1b* in osteoarthritic synovium, we established post-traumatic OA model in mice by DMM and performed immunohistochemistry staining of their knee joint. The results showed that the positive cell rate of ACADL and ADH1B were significantly lower in OA synovium, respectively (Figures 7A–D). Consistently, ACADL and ADH1B protein levels were lower in OA synovium revealed by Western blot analysis (Figure 7E).

## 4 Discussion

OA is known to be closely associated with metabolic disorders. Previous studies have demonstrated that an abnormal KBM in chondrocytes promotes OA progression by disrupting energy homeostasis (32). In this study, for the first time, we integrated multi-omics data and machine learning algorithms to systematically identify the KBM-related genes *ACADL* and *ADH1B* in synovial tissues as key biomarkers for OA diagnosis, further validating their decreased expression in OA animal models. The clinical nomogram model constructed using these two genes exhibited excellent predictive efficacy (area under the curve = 0.99), highlighting the potential of these genes as therapeutic targets for OA.

*ACADL* encodes the rate-limiting enzyme of mitochondrial fatty acid β-oxidation, governing ATP generation to sustain the primary energy supply under stress (33, 34). In our study, both bioinformatic analyses and animal models demonstrated significant downregulation of *ACADL* expression in the OA synovium, concurrent with lipid droplet accumulation. This is similar to the lipid accumulation observed in chondrocytes (35, 36). PPI network analysis revealed that *ACADL* formed a metabolic module with HADH, suggesting that *ACADL* may impair ketone body synthesis under hypoxic conditions by reducing acetyl-CoA production. This could result in a compromised energy supply to synoviocytes, as well as an elevation of lipid byproducts, including inflammatory factors. Consequently, decreased *ACADL* may contribute to the pathogenesis of pain and joint swelling in patients by enhancing neutrophil chemotaxis and macrophage activation in the synovium, accompanied by the release of inflammatory factors. In the literature, Chung et al. confirmed that *ACADL* deficiency in type II alveolar epithelial cells inhibits the production of the neutrophil chemokine C-X-C motif chemokine ligand 2 by impairing mitochondrial long-chain fatty acid β-oxidation, consequently attenuating alveolar inflammation in acute lung injury (37). Further, Zhang et al. verified that the marine cyclopeptide Stylissatin A promotes fatty acid accumulation in macrophages by inhibiting *ACADL*-mediated fatty acid β-oxidation, thereby blocking NF-κB signaling and inducible nitric oxide synthase expression to exert anti-inflammatory effects (38). These contradictory tissue-specific roles of *ACADL* require further in-depth investigation.

*ADH1B*, a member of the alcohol dehydrogenase family, is highly expressed in the adipose tissue and exerts antioxidant effects in OA (39, 40). Consistent with this knowledge, we identified *ADH1B* downregulation as a signature of OA, which may impair the NAD^+^/NADH balance, leading to the accumulation of reactive oxygen species. The accumulation of *ADH1B* in synoviocytes may activate the NF-κB and MAPK pathways, inducing massive secretion of proinflammatory cytokines and chemokines. This drives the recruitment of immune cells, including neutrophils and macrophages, into the joint cavity, thereby forming a vicious cycle of inflammation. Reactive oxygen species can also directly or indirectly activate matrix metalloproteinases and aggrecanases, which excessively degrade cartilage collagen and proteoglycans, ultimately resulting in joint destruction. Furthermore, a significant negative correlation was identified between *ADH1B* expression and macrophage infiltration. Importantly, prior research has shown that inhibition of M1 macrophage polarization in the synovium can delay OA progression, possibly due to the regulation of the secretion of inflammatory factors and remodeling of the local immune microenvironment of the joint (41–43). These results indicate that modulation of the local immune microenvironment to restore articular homeostasis is viable.

Acetaminophen is a first-line analgesic for OA. However, its clinical utility is limited by its dose-dependent hepatotoxicity (44). Treatment with fomepizole can mitigate this risk by inhibiting CYP2E1-mediated NAPQI formation and reducing JNK-associated oxidative stress (42). When combined with N-acetylcysteine, this represents a promising strategy for managing acetaminophen overdoses (42). On the other hand, progesterone acts as a potential disease-modifying agent for OA, particularly in postmenopausal women, by promoting chondrocyte survival and exerting anti-inflammatory effects (45). However, their clinical translations require further validation through more rigorous preclinical studies and systematic clinical trials to fully elucidate the associated therapeutic potential and safety profiles.

Collectively, this study proposes that the novel biomarkers *ACADL* and *ADH1B* could serve as objective and efficient diagnostic indicators of OA. Candidate compounds, such as progesterone and fomepizole, have laid a theoretical foundation for the development of OA-modifying drugs. Regarding the mechanism, this study further clarified how KBM affects joint homeostasis by clarifying the metabolism-immunity axis of the OA synovium. Future research is needed to explore the role of the abnormal synovium in driving OA development and to evaluate the potential of targeted interventions to alleviate OA synovial metabolic disorders, thereby more effectively relieving patient symptoms and improving prognosis.

## 5 Conclusion

Bioinformatics analysis and animal experiments confirmed that *ACADL* and *ADH1B* are KBM-related genes downregulated in OA synovial tissues. They exhibit excellent clinical predictive value and are significantly associated with the infiltration of various immune cells. Progesterone and fomepizole, as the predicted drugs targeting *ACADL* and *ADH1B* respectively, hold potential for OA treatment.

## Data Availability

The raw data supporting the conclusions of this article will be made available by the authors, without undue reservation.

## Footnotes

1
http://ncbi.nlm.nih.gov/geo/

2
https://gsea-msigdb.org/gsea/msigdb

3
https://string-db.org/

4
https://gsea-msigdb.org/gsea/msigdb

5
https://genemania.org/

6
https://dgidb.org/

7
https://ctdbase.org/

8
https://pubchem.ncbi.nlm.nih.gov/

9
https://cadd.labshare.cn/cb-dock2/php/index.php

## Supplementary material

SUPPLEMENTARY TABLE 1 The list containing the genes related to ketone body metabolism.

SUPPLEMENTARY TABLE 2 GO enrichment of biological processes by analyzing the candidate genes.

SUPPLEMENTARY TABLE 3 GO enrichment of cellular components by analyzing the candidate genes.

Supplementary Table 4 GO enrichment of molecular functions by analyzing the candidate genes.

SUPPLEMENTARY TABLE 5 KEGG pathway enrichment by analyzing the candidate genes.

SUPPLEMENTARY TABLE 6 The list of ACADL-enriched biological processes.

SUPPLEMENTARY TABLE 7 The list of ADH1B-enriched biological processes.

SUPPLEMENTARY TABLE 8 The statistical matrix of cross-cell-type correlation analysis of immune cell subtypes.

SUPPLEMENTARY TABLE 9 The statistical matrix demonstrating the correlation analysis between ACADL/ADH1B and immune cell subtypes.

## References

[1] Courties A Kouki I Soliman N Mathieu S Sellam J. Knee effusions, popliteal cysts, and synovial thickening: association with knee pain in osteoarthritis. *Osteoarthritis Cartilage.* (2024) 32:1397–404. 10.1016/j.joca.2024.07.014 39103081

[2] Steinmetz JD Culbreth GT Haile LM Rafferty Q Lo J Fukutaki KG Global, regional, and national burden of osteoarthritis, 1990–2020 and projections to 2050: a systematic analysis for the Global Burden of Disease study 2021. *Lancet Rheumatol.* (2023) 5:e508–22. 10.1016/s2665-9913(23)00163-7 37675071 PMC10477960

[3] Sanchez-Lopez E Coras R Torres A Lane NE Guma M. Synovial inflammation in osteoarthritis progression. *Nat Rev Rheumatol.* (2022) 18:258–75. 10.1038/s41584-022-00749-9 35165404 PMC9050956

[4] Guo S-K Suo J Huang Y Yin X Wang J Li L Therapeutic circRNA aptamer alleviates PKR-associated osteoarthritis. *Sci. Bull.* (2025) 70:2232–6. 10.1016/j.scib.2025.02.027 40021383

[5] Katz JN Arant KR Loeser RF. Diagnosis and treatment. *JAMA.* (2021) 325:568. 10.1001/jama.2020.22171 33560326 PMC8225295

[6] Slomski A. Physical therapy outperforms injections for knee osteoarthritis. *JAMA.* (2020) 323:2453. 10.1001/jama.2020.9155 32573664

[7] Ayral X Pickering EH Woodworth TG Mackillop N Dougados M. Synovitis: a potential predictive factor of structural progression of medial tibiofemoral knee osteoarthritis – results of a 1 year longitudinal arthroscopic study in 422 patients. *Osteoarthritis Cartilage.* (2005) 13:361–7. 10.1016/j.joca.2005.01.005 15882559

[8] Roemer FW Kassim Javaid M Guermazi A Thomas M Kiran A Keen R Anatomical distribution of synovitis in knee osteoarthritis and its association with joint effusion assessed on non-enhanced and contrast-enhanced MRI. *Osteoarthritis Cartilage.* (2010) 18:1269–74. 10.1016/j.joca.2010.07.008 20691796

[9] Klein-Wieringa IR De Lange-Brokaar BJE Yusuf E Andersen SN Kwekkeboom JC Kroon HM Inflammatory cells in patients with endstage knee osteoarthritis: a comparison between the synovium and the infrapatellar fat pad. *J Rheumatol.* (2016) 43:771–8. 10.3899/jrheum.151068 26980579

[10] Qin J Huang X Gou S Zhang S Gou Y Zhang Q Ketogenic diet reshapes cancer metabolism through lysine β-Hydroxybutyrylation. *Nat Metab.* (2024) 6:1505–28. 10.1038/s42255-024-01093-w 39134903

[11] Cao X Cui Z Ding Z Chen Y Wu S Wang X An osteoarthritis subtype characterized by synovial lipid metabolism disorder and fibroblast-like synoviocyte dysfunction. *J Orthopaedic Transl.* (2022) 33:142–52. 10.1016/j.jot.2022.02.007 35330945 PMC8919236

[12] Kong G Wang J Li R Huang Z Wang L. Ketogenic diet ameliorates inflammation by inhibiting the NLRP3 inflammasome in osteoarthritis. *Arthritis Res Ther.* (2022) 24:113. 10.1186/s13075-022-02802-0 35585627 PMC9116003

[13] Zhuang H Ren X Zhang Y Li H Zhou P. β-Hydroxybutyrate enhances chondrocyte mitophagy and reduces cartilage degeneration in osteoarthritis via the HCAR2/AMPK/PINK1/parkin pathway. *Aging Cell.* (2024) 23:e14294. 10.1111/acel.14294 39126207 PMC11561673

[14] Ritchie ME Phipson B Wu D Hu Y Law CW Shi W Limma powers differential expression analyses for RNA-sequencing and microarray studies. *Nucleic Acids Res.* (2015) 43:e47–47. 10.1093/nar/gkv007 25605792 PMC4402510

[15] Gustavsson EK Zhang D Reynolds RH Garcia-Ruiz S Ryten M. Ggtranscript: an R package for the visualization and interpretation of transcript isoforms using Ggplot2. *Bioinformatics.* (2022) 38:3844–6. 10.1093/bioinformatics/btac409 35751589 PMC9344834

[16] Gu Z Hübschmann D. Make interactive complex heatmaps in R. *Bioinformatics.* (2022) 38:1460–2. 10.1093/bioinformatics/btab806 34864868 PMC8826183

[17] Zhou J Huang J Li Z Song Q Yang Z Wang L Identification of aging-related biomarkers and immune infiltration characteristics in osteoarthritis based on bioinformatics analysis and machine learning. *Front Immunol.* (2023) 14:1168780. 10.3389/fimmu.2023.1168780 37503333 PMC10368975

[18] Wu T Hu E Xu S Chen M Guo P Dai Z clusterProfiler 4.0: a universal enrichment tool for interpreting omics data. *Innovation.* (2021) 2:100141. 10.1016/j.xinn.2021.100141 34557778 PMC8454663

[19] Wang L Wang D Yang L Zeng X Zhang Q Liu G Cuproptosis related genes associated with Jab1 shapes tumor microenvironment and pharmacological profile in nasopharyngeal carcinoma. *Front Immunol.* (2022) 13:989286. 10.3389/fimmu.2022.989286 36618352 PMC9816571

[20] Shannon P Markiel A Ozier O Baliga NS Wang JT Ramage D Cytoscape: a software environment for integrated models of biomolecular interaction networks. *Genome Res.* (2003) 13:2498–504. 10.1101/gr.1239303 14597658 PMC403769

[21] Zhou H Xin Y Li S. A diabetes prediction model based on boruta feature selection and ensemble learning. *BMC Bioinformatics.* (2023) 24:224. 10.1186/s12859-023-05300-5 37264332 PMC10236811

[22] Hou N Li M He L Xie B Wang L Zhang R Predicting 30-days mortality for MIMIC-III patients with sepsis-3: a machine learning approach using XGboost. *J Transl Med.* (2020) 18:462. 10.1186/s12967-020-02620-5 33287854 PMC7720497

[23] Gao C-H Yu G Cai P. ggVennDiagram: an intuitive, easy-to-use, and highly customizable R package to generate venn diagram. *Front Genet.* (2021) 12:706907. 10.3389/fgene.2021.706907 34557218 PMC8452859

[24] Xu J Yang T Wu F Chen T Wang A Hou S. A nomogram for predicting prognosis of patients with cervical cerclage. *Heliyon.* (2023) 9:e21147. 10.1016/j.heliyon.2023.e21147 37885715 PMC10598483

[25] Sui Z Wu X Du L Wang H Yuan L Zhang JV Characterization of the immune cell infiltration landscape in esophageal squamous cell carcinoma. *Front. Oncol.* (2022) 12:879326. 10.3389/fonc.2022.879326 35875070 PMC9300817

[26] Robin X Turck N Hainard A Tiberti N Lisacek F Sanchez J-C pROC: an open-source package for R and S+ to analyze and compare ROC curves. *BMC Bioinformatics.* (2011) 12:77. 10.1186/1471-2105-12-77 21414208 PMC3068975

[27] Robles-Jimenez LE Aranda-Aguirre E Castelan-Ortega OA Shettino-Bermudez BS Ortiz-Salinas R Miranda M Worldwide traceability of antibiotic residues from livestock in wastewater and soil: a systematic review. *Animals.* (2021) 12:60. 10.3390/ani12010060 35011166 PMC8749557

[28] Yu G Wang L-G Han Y He Q-Y. clusterProfiler: an R package for comparing biological themes among gene clusters. *OMICS J Integr Biol.* (2012) 16:284–7. 10.1089/omi.2011.0118 22455463 PMC3339379

[29] Aran D Hu Z Atul JB. xCell: digitally portraying the tissue cellular heterogeneity landscape. *Genome Biol.* (2017) 18:220. 10.1186/s13059-017-1349-1 29141660 PMC5688663

[30] Yao Z Han J Wu J Li M Chen R Jian M Deciphering the multidimensional impact of IGFBP1 expression on cancer prognosis, genetic alterations, and cellular functionality: a comprehensive pan-cancer analysis. *Heliyon.* (2024) 10:e37402. 10.1016/j.heliyon.2024.e37402 39309809 PMC11416238

[31] Glasson SS Blanchet TJ Morris EA. The surgical destabilization of the medial meniscus (DMM) model of osteoarthritis in the 129/SvEv mouse. *Osteoarthritis Cartil.* (2007) 15:1061–9. 10.1016/j.joca.2007.03.006 17470400

[32] Cai Z Zhang Z Leng J Xie M Zhang K Zhang J β-Hydroxybutyrate ameliorates osteoarthritis through activation of the ERBB3 signaling pathway in mice. *J Bone Mineral Res.* (2024) 40:140–53. 10.1093/jbmr/zjae176 39498503

[33] Wang C Qiao S Zhao Y Tian H Yan W Hou X The KLF7/PFKL/ACADL axis modulates cardiac metabolic remodelling during cardiac hypertrophy in male mice. *Nat Commun.* (2023) 14:959. 10.1038/s41467-023-36712-9 36810848 PMC9944323

[34] Chiba T Oda A Zhang Y Pfister K Bons J Bharathi SS Loss of long-chain Acyl-CoA dehydrogenase protects against acute kidney injury. *JCI Insight.* (2025) 10:e186073. 10.1172/jci.insight.186073 39932791 PMC11949023

[35] Cai J Chen T Jiang Z Yan J Ye Z Ruan Y Bulk and single-cell transcriptome profiling reveal extracellular matrix mechanical regulation of lipid metabolism reprograming through YAP/TEAD4/ACADL axis in hepatocellular carcinoma. *Int J Biol Sci* (2023) 19:2114–31. 10.7150/ijbs.82177 37151879 PMC10158031

[36] Liu H Witzigreuter L Sathiaseelan R Agbaga M Brush RS Stout MB Knee effusions, popliteal cysts, and synovial thickening: association with knee pain in osteoarthritis. *J Orthopaedic Res.* (2022) 40:2771–9. 10.1002/jor.25322 35279877 PMC9647658

[37] Chung K-P Cheng C-N Chen Y-J Hsu C-L Huang Y-L Hsieh M-S Alveolar epithelial cells mitigate neutrophilic inflammation in lung injury through regulating mitochondrial fatty acid oxidation. *Nat Commun.* (2024) 15:7241. 10.1038/s41467-024-51683-1 39174557 PMC11341863

[38] Zhang M Sunaba T Sun Y Shibata T Sasaki K Isoda H Acyl-CoA dehydrogenase long chain (ACADL) is a target protein of stylissatin A, an anti-inflammatory cyclic heptapeptide. *J Antibiot.* (2020) 73:589–92. 10.1038/s41429-020-0322-5 32439989

[39] Morales LD Cromack DT Tripathy D Fourcaudot M Kumar S Curran JE Further evidence supporting a potential role for ADH1B in obesity. *Sci Rep.* (2021) 11:1932. 10.1038/s41598-020-80563-z 33479282 PMC7820614

[40] Gautheron J Elsayed S Pistorio V Lockhart S Zammouri J Auclair M ADH1B, the adipocyte-enriched alcohol dehydrogenase, plays an essential, cell-autonomous role in human adipogenesis. *Proc Natl Acad Sci U S A.* (2024) 121:e2319301121. 10.1073/pnas.2319301121 38838011 PMC11181076

[41] Lv Z Xu X Sun Z. TRPV1 alleviates osteoarthritis by inhibiting M1 macrophage polarization via Ca2+/CaMKII/Nrf2 signaling pathway. *Cell Death Dis.* (2021) 12:504. 10.1038/s41419-021-03792-8 34006826 PMC8131608

[42] Xu Z Peng B Kang F Zhang W Xiao M Li J The roles of drug metabolism-related ADH1B in immune regulation and therapeutic response of ovarian cancer. *Front Cell Dev Biol.* (2022) 10:877254. 10.3389/fcell.2022.877254 35756990 PMC9218672

[43] Li H Yuan Y Zhang L Xu C Xu H Chen Z. Reprogramming macrophage polarization, depleting ROS by astaxanthin and thioketal-containing polymers delivering rapamycin for osteoarthritis treatment. *Adv Sci.* (2024) 11:e2305363. 10.1002/advs.202305363 38093659 PMC10916582

[44] Conaghan PG Arden N Avouac B Migliore A Rizzoli R. Safety of paracetamol in osteoarthritis: what does the literature say? *Drugs Aging.* (2019) 36:7–14. 10.1007/s40266-019-00658-9 31073920 PMC6509082

[45] Gilmer G Iijima H Hettinger ZR Jackson N Bergmann J Bean AC Menopause-induced 17β-Estradiol and progesterone loss increases senescence markers, matrix disassembly and degeneration in mouse cartilage. *Nat Aging.* (2025) 5:65–86. 10.1038/s43587-024-00773-2 39820791 PMC12999526

